# Characterization of Psychotic Experiences in Adolescence Using the Specific Psychotic Experiences Questionnaire: Findings From a Study of 5000 16-Year-Old Twins

**DOI:** 10.1093/schbul/sbt106

**Published:** 2013-09-23

**Authors:** Angelica Ronald, Dominika Sieradzka, Alastair G. Cardno, Claire M. A. Haworth, Philip McGuire, Daniel Freeman

**Affiliations:** ^1^Centre for Brain and Cognitive Development, Birkbeck, University of London, London, UK;; ^2^Academic Unit of Psychiatry and Behavioural Sciences, University of Leeds, Leeds, UK;; ^3^Social Genetic and Developmental Psychiatry Centre, Institute of Psychiatry, King’s College London, London, UK;; ^4^Institute of Psychiatry, King’s College London, London, UK;; ^5^Department of Psychiatry, University of Oxford, Oxford, UK

**Keywords:** paranoia, hallucinations, grandiosity, anhedonia, adolescence, schizophrenia

## Abstract

We aimed to characterize multiple psychotic experiences, each assessed on a spectrum of severity (ie, quantitatively), in a general population sample of adolescents. Over five thousand 16-year-old twins and their parents completed the newly devised Specific Psychotic Experiences Questionnaire (SPEQ); a subsample repeated it approximately 9 months later. SPEQ was investigated in terms of factor structure, intersubscale correlations, frequency of endorsement and reported distress, reliability and validity, associations with traits of anxiety, depression and personality, and sex differences. Principal component analysis revealed a 6-component solution: paranoia, hallucinations, cognitive disorganization, grandiosity, anhedonia, and parent-rated negative symptoms. These components formed the basis of 6 subscales. Correlations between different experiences were low to moderate. All SPEQ subscales, except Grandiosity, correlated significantly with traits of anxiety, depression, and neuroticism. Scales showed good internal consistency, test-retest reliability, and convergent validity. Girls endorsed more paranoia, hallucinations, and cognitive disorganization; boys reported more grandiosity and anhedonia and had more parent-rated negative symptoms. As in adults at high risk for psychosis and with psychotic disorders, psychotic experiences in adolescents are characterized by multiple components. The study of psychotic experiences as distinct dimensional quantitative traits is likely to prove an important strategy for future research, and the SPEQ is a self- and parent-report questionnaire battery that embodies this approach.

## Introduction

Psychotic disorders such as schizophrenia and bipolar disorder typically begin in early adulthood sometime after the age of 18 years.^1^ This makes studying psychotic experiences in adolescence, just prior to the most common age of onset of clinical disorder, especially interesting. Children and adolescents who display psychotic experiences have an elevated risk of developing psychosis.^2^ Individuals who have psychotic experiences share some of the same risk factors as those known to be associated with psychotic disorders.^3,4^ The last decade has seen increasing interest in the development of clinical interventions for individuals at high risk of psychosis.^5,6^

### Studies of Psychotic Experiences in Adolescents

One population-based adolescent study, using a Spanish sample of seven hundred and seventy-seven 13- to 17-year olds, has assessed both positive and negative psychotic experiences with a measure that takes to some degree a dimensional approach.^7^ A Spanish version of the Community Assessment of Psychotic Experiences (CAPE) was used. Principal component analysis suggested that both positive and negative psychotic experiences, which were factor analyzed separately, are made up of multiple factors. While the sample size was adequate for psychometric analyses, greater power—especially for studying the less common experiences—would be achieved with a much larger sample. CAPE uses items with content adapted directly from clinical scales. Notably, a prospective longitudinal study in Munich has also been conducted on an adolescent and young adult sample (14–24 years). The sample was assessed on both positive and negative psychotic experiences although reported findings have tended to focus on either a total psychosis scale^8^ or on dichotomized data^9^ (as opposed to studying specific quantitative dimensions). A spectrum of severity in item content from the mild to the clinical is needed to assess experiences truly dimensionally. For example, in assessing paranoia, one would wish to study a spectrum of content from mild suspicions that others have an interest in the person all the way to fears of conspiracies.

Some strong data on positive psychotic experiences in adolescents come from 2 general population samples in the Netherlands of 16-year olds showing that positive psychotic experiences fall into 5 factors (hallucinations, paranoia, grandiosity, delusions, and paranormal beliefs), when assessed using the 20-item self-rated CAPE positive psychotic experiences scale.^10^ CAPE was also completed by eight hundred and seventy-five 15-year-old adolescents from Melbourne, and 4 factors were reported (bizarre experiences, perceptual abnormalities, persecutory ideas, and magical thinking).^11^ This finding was replicated using the same measure in a sample including both adolescents and young adults: again a 4-factor solution was found (magical thinking replaced grandiosity).^12^ CAPE provides replicable findings; its brevity, while practical for use on large cohorts, and its derivation from clinical instruments limit the range of mild to severe experiences that can be detected.

Positive psychotic experiences, measured using a single “total” scale, have been assessed in at least 4 longitudinal child or adolescent cohorts.^2,13–15^ Other studies have used a handful of items about positive psychotic experiences in general population adolescent samples.^16,17^ Finally, some surveys have focused on a single item relating to auditory hallucinations in studies of adolescent cohorts.^18^

Research on negative symptoms in adolescent general population samples is less common. One study investigated social anhedonia in a representative sample of 18-year olds in terms of its predictiveness for concurrent and later schizophrenia-spectrum personality disorder characteristics.^19^ We are not aware of any representative general population samples of adolescents that have been assessed on multiple positive and negative psychotic experiences using truly dimensional scales.

### Current Study

To capture naturally occurring quantitative variation in specific psychotic experiences from the low extreme to the high extreme, for this study the authors developed a new measure battery, the Specific Psychotic Experiences Questionnaire (SPEQ). The aim was to assess the key types of specific psychotic experiences traditionally associated with psychosis: paranoia, hallucinations, cognitive disorganization, grandiosity, anhedonia (all self-rated), and parent-rated negative symptoms. Existing measures were employed for individual subscales and adapted for an adolescent sample.

The research aims were, first, to investigate the factor structure of specific psychotic experiences. It was hypothesized that positive and negative psychotic experiences would fall into separate components, with positive psychotic experiences best modeled with multiple components, as per previous findings.^7^ It was also hypothesized that negative symptoms would comprise at least 2 components. A previous review reported that the most consistent 2 domains found in negative symptoms within schizophrenia were diminished expression and anhedonia.^20^ In light of previous mixed findings, exploratory rather than confirmatory factor analysis was employed.

The second aim was to assess the degree to which specific types of psychotic experiences were correlated. Based on past research, it was hypothesized that SPEQ subscales would not be characterized by high interscale correlations but by modest or non-significant correlations. Third, the frequency of psychotic experiences was assessed and compared with past findings from singletons.^4,18,21^ It was hypothesized that these experiences would be regular (ie, monthly/weekly or more frequent) occurrences in a minority of the sample and that the frequency would be higher than that of psychotic disorders.^21^ Distress associated with psychotic experiences was also measured.

The fourth aim was to test the degree of association between SPEQ and another adolescent psychosis experiences scale, as well as associations with traits of anxiety, depression, and general forms of personality. These results would be informative regarding construct validity. It was hypothesized that strong significant correlations would be shown between SPEQ subscales (with exception of grandiosity) and traits of anxiety, depression and neuroticism, in line with previous findings from clinical and community samples,^7,12,22–24^ but that there would also be variance independent of these traits.

SPEQ subscales were also tested for mean sex differences. Finally, because the main sample used was a twin sample, mean scores in twins and singletons were compared to test the generalizability of these data to nontwin populations.

## Methods

### Participants

The Longitudinal Experiences And Perceptions (LEAP) study focuses on specific psychotic experiences in adolescence. The sample was drawn from the Twins Early Development Study (TEDS), a general population study of twins born in England and Wales between 1994–1996.^25^ TEDS has full ethical approval. In 1994–1996, TEDS contacted a sample of 16 302 families who had recently had twins, of whom 13 488 families responded with a written consent form. Families were not subsequently contacted for LEAP if they had withdrawn from TEDS, were inactive, had known address problems, or were special cases, most notably medical exclusions.

*Singleton (Pilot) Sample.* Seventy families were contacted to participate in the pilot study, of whom 52 responded (74%). The pilot sample consisted of 17-year-old singletons (*M* = 17.03 years; SD = 0.55; years; 39% male). These were full siblings of twins in TEDS (*N* = 45), and 7 individuals unrelated to TEDS families. This sample was used for twin-singleton comparisons and for piloting.

*LEAP twin sample, phase 1*. A total of 10 874 TEDS families were contacted and invited to participate in the LEAP study. Of those contacted, 5076 (47%) parents and 5059 (47%) twin pairs provided data (*M* = 16.32 years; SD = 0.68 years). Individuals were excluded (*N* = 316 families) if they did not provide consent at first contact (when TEDS was started) or for this study, if they had severe medical disorder, if they had experienced severe perinatal complications or if zygosity was unknown. Sample after exclusions (*N* = 4743 families) was 45% male. Online supplementary table S1 presents demographic information comparing the participating and nonparticipating families who were contacted.

*LEAP twin sample, phase 2*. In phase 2, one thousand seven hundred and seventy-three of the same families were invited to participate again on average 9 months later. Data from 1464 families (83%) were obtained from both parents and twins (*M* = 17.06 years; SD = 0.88 years).

### Measures

#### Specific Psychotic Experiences Questionnaire.

Six types of psychotic experiences were assessed: paranoia, hallucinations, cognitive disorganization, grandiosity, anhedonia, all via self-report, and negative symptoms via parent report. The online supplementary appendix provides further details. *Paranoia* was assessed with 15 items from the Paranoia Checklist.^21^ *Hallucinations* were assessed with 9 items from the Cardiff Anomalous Perceptions Scale.^26^ *Cognitive disorganization* was assessed with 11 items from the short Oxford-Liverpool Inventory of Feelings and Experiences.^27^ *Grandiosity* was assessed with items from the “Myself” subscale of Cognition Checklist for Mania-Revised,^28^ the Peters and colleagues’ Delusions Inventory,^29^ and items developed from clinical case studies. *Anhedonia* was assessed with 10 items from the anticipatory pleasure subscale of the Temporal Experience of Pleasure Scale^30^ (the scale was reversed so that higher scores indicated more anhedonia). *Parent-rated negative symptoms* were assessed with 10 items devised from the Scale for the Assessment of Negative Symptoms.^31^ For negative symptoms, both self-report and rater (parent) report were employed, in line with recent recommendations.^32^

Finally, *distress* was assessed with an item at the end of each of the paranoia, hallucinations, cognitive disorganization, and grandiosity subscales.

### Additional Measures

Anxiety was assessed using an age-adapted version of the parent-rated Anxiety-Related Behaviors Questionnaire (younger age versions are described elsewhere)^33^ and the self-rated Childhood Anxiety Sensitivity Index.^34^ Depression was assessed by self-ratings and parent ratings on the Short Moods and Feelings Questionnaire.^35^ Personality was assessed via self-report on a published scale.^36^ The psychosis-like symptoms (PLIKS) measure from the Avon Longitudinal Study of Parents and Children 16-year child questionnaire was included in phase 2 to test SPEQ’s construct validity.^37^

### Analyses

One twin per pair (selected randomly for birth order) was included for psychometric analyses.

#### Principal Component Analyses.

Principal component analyses (PCA) with no rotation, and with Varimax and Oblimin rotations, were conducted on SPEQ items. Factors were extracted using a scree plot, and cutoff criterion for item loadings was fixed at ±.30.

#### SPEQ Scale Construction.

Scales were developed on the basis of PCA results (see Results). Items in each subscale were summed and converted into scores as a proportion of the total possible score given the number of items completed (which had to be more than half). Due to the moderate skew of 4 subscales (paranoia, hallucinations, grandiosity, and parent-rated negative symptoms), transformed scales, using log10(1 + variable) formulae, were employed for *t* tests.

#### *t* Tests.

Two-tailed independent *t* tests were conducted to explore mean group differences. Where Levene’s test was significant, *P* values for corrected degrees of freedom (*df*) were reported. Bonferroni corrections were applied.

#### Correlations.

Spearman’s correlations were used to assess SPEQ intersubscale correlations and correlations with anxiety, depression, personality, and PLIKS.

#### Validity.

Construct validity was assessed in terms of the PCA supporting the separation of subscales and a positive association shown with neuroticism, anxiety, and depression. Content validity was assessed via expert clinical opinion to judge the suitability of items for measuring adolescent psychotic experiences (by A.G.C., D.F., and P.M.). Finally, validity was also assessed in terms of agreement with a second known measure of adolescent PLIKS.^37^ A quantitative score, based on responses on 10 PLIKS items asking about the presence of positive PLIKS, was constructed for the LEAP phase 2 sample and correlated with SPEQ subscales. Additionally, a binary variable was computed from PLIKS items to identify individuals who reported “definitely” having any positive PLIKS.^37^ Independent samples *t* tests compared the PLIKS-defined groups with and without PLIKS on SPEQ subscales.

#### Twin-Singleton Comparisons.

Paired within-family *t* tests compared means between singleton siblings and each cotwin per family. Grandiosity subscale was excluded due to incomparable items across twins and singletons (see online supplementary appendix).

## Results

### Structure of Psychotic Experiences

#### Principal Component Analyses.

**Fig. 1.**
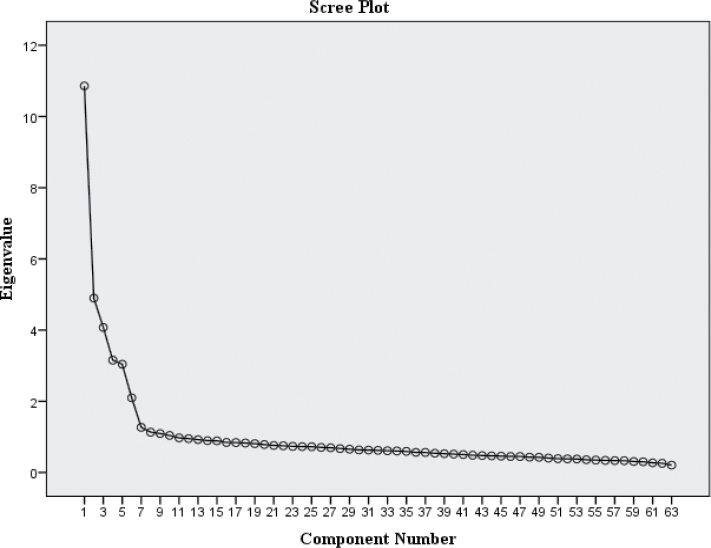
Scree plot from principal component analysis.

Figure 1 presents the scree plot, which suggested a 6-component solution: paranoia, hallucinations, cognitive disorganization, grandiosity, anhedonia, and parent-rated negative symptoms. Correlations between components were low to moderate (.00–.43) with only 3 correlations out of 15 being >.3. For this reason, Varimax rotation was reported.

The 6 components explained 44.6% of the variance, with component 1 accounting for 12.5% postextraction and rotation. The other 5 components accounted for 5.6%–7.3% variance. Factor loadings had a clear structure outlined in online supplementary table S2, with each dimension of psychotic experiences loading onto a separate component. Subscales were created with items as per their membership to each component in online supplementary table S2.

### Presence of Psychotic Experiences

#### Frequency of Endorsement.

SPEQ was not designed to assess prevalence of discrete symptoms but rather to measure quantitative dimensions. Nevertheless, mean scores in table 1 show the average ratings in the twin sample. In terms of the proportion of individuals having frequent experiences, across all hallucination items, 5.9%–23.2% reported having Hallucinations monthly or more often. In terms of vocal hallucinations, “*How often do you hear voices commenting on what you’re thinking or doing?*” was endorsed “*Not at all*” by 85.1%, “*Rarely*” by 8.9%, “*Once a month*” by 2.5%, “*Once a week*” by 1.2%, “*Several times a week*” by 0.9%, and “*Daily*” by 1.3%. As such, this item was endorsed between “*Once a month*” and “*Daily*” by 5.9% of the sample, which is similar to the median prevalence reported in meta-analysis (7.5%).^18^

**Table 1.** Descriptives

	Paranoia	Hallucinations	Cognitive Disorganization	Grandiosity	Anhedonia	Parent-Rated Negative Symptoms
*N*	4731	4739	4732	4735	4735	4746
Mean	12.14	4.62	3.95	5.31	16.36	2.82
SD	10.58	5.94	2.85	4.41	7.93	3.90
Variance	112.01	35.33	8.11	19.47	62.83	15.20
Observed range	0–72	0–45	0–11	0–24	0–50	0–30
Median	10.00	2.00	4.00	4.00	16.00	1.00
Mode	.00	.00	3.00	2.00	15.00	.00
Skewness	1.55	2.05	.45	1.18	.49	2.36
Kurtosis	3.14	5.25	−.64	1.43	.05	7.15
Cronbach’s α	.93	.87	.77	.85	.78	.85
Test-retest reliability (*N* = 1427–1437)^a^	.66**	.65**	.74**	.66**	.70**	.68**

*Note*: Cronbach’s α did not increase following removal of any items.

^a^Spearman correlation between phase 1 and phase 2, an average 9-month gap.

**P < .001.

Across paranoia, 1%–23% reported having paranoid thoughts as weekly or more often, which was a slightly lower range than that reported by adults.^21^ An example paranoia item, “*How often have you thought I need to be on my guard against others*,” was endorsed: “*Not at all*” 26.7%, “*Rarely*” 48.1%, “*Once a month*” 9.9%, “*Once a week*” 7.4%, “*Several times a week*” 5.1%, and “*Daily*” 2.8%. An example of a less common paranoia item, “*I am under threat from others*,” was endorsed: “*Not at all*” 75.1%, “*Rarely*” 20.6%, “*Once a month*” 2.4%, “*Once a week*” 1.0%, “*Several times a week*” 0.6%, and “*Daily*” 0.3%.

The other psychotic experiences did not have response scales in terms of frequency. Cognitive disorganization items were endorsed as “*Yes*” by 20.2%–51.3% of the sample. Grandiosity items were endorsed as “*A great deal*” or “*Completely*” by 7.1%–31.0% of the sample. Anhedonia items (phrased in the direction of Hedonia, see online supplementary appendix), were rated as “*Moderately false to me*” or “*Very false to me*” by 1.7%–37.6% of the sample. Parent-rated negative symptom items were rated as “*Mainly true*” or “*Definitely true*” for 1.9%–5.8% of the sample (when including “*Somewhat True*” the range increased to 10.4%–33.1% of the sample).

#### Distress.

Online supplementary table S3 shows that 5.7% reported being quite or very distressed by cognitive disorganization. Paranoia was second highest, with 4.2% reported being quite or very distressed, and <2% reported finding grandiosity and hallucinations quite or very distressing.

#### Descriptives, Reliability, and Sex Differences.

Descriptive statistics are reported in table 1. SPEQ subscales showed good to excellent internal consistency (Cronbach’s α = .77–.93). Test-retest reliability across on average 9 months was *r =* .65–.74 (all *P* < .001).

Females scored significantly higher than males on paranoia, hallucinations, and cognitive disorganization; males scored significantly higher than females on grandiosity, anhedonia, and parent-rated negative symptoms (based on *P* < .008) (see online supplementary table S4).

#### Correlations Between SPEQ Subscales.

Table 2 presents correlations between SPEQ subscales. The median correlation was ±.14 and correlations ranged from *r* = ±.00–.45. The 3 highest correlations (all *r* > .4) were between paranoia and hallucinations (*r* = .45, *P* < .001), paranoia and cognitive disorganization (*r* = .40, *P* < .001), and hallucinations and cognitive disorganization (*r* = .43, *P* < .001). Despite parent-rated negative symptoms having a different rater to the other (self-rated) subscales, they correlated significantly (*r* = ±.13–.24, all *P* < .001) with all SPEQ subscales except for grandiosity. Grandiosity and anhedonia showed low to modest correlations with other SPEQ subscales (*r* = ±.00–.18). Online supplementary table S5 presents correlations split by sex.

**Table 2.** Interscale Correlations Between Psychotic Experiences in Adolescence

	Paranoia	Hallucinations	Cognitive Disorganization	Grandiosity	Anhedonia
Hallucinations	.45**	—	—	—	—
Cognitive disorganization	.40**	.43**	—	—	—
Grandiosity	.10**	.18**	.02	—	—
Anhedonia	.06**	.00	.01	−.17**	—
Parent-rated negative symptoms	.14**	.13**	.24**	−.02	.14**

*Note*: *N* = 4699–4733.

**P < .001.

#### Construct Validity.

Correlations between the PLIKS quantitative score and SPEQ positive subscales were the following: hallucinations *r* = .60, paranoia *r* = .48, cognitive disorganization *r* = .41, grandiosity *r* = .27 (all *P* < .001). Individuals who scored as “affected” on the PLIKS binary outcome score had significantly more psychotic experiences on SPEQ subscales than the “unaffected” group except anhedonia (*P* < .008; see online supplementary table S6).

#### Twin-Singleton Comparisons.

There was a trend for singleton siblings to report more psychotic experiences than their twin siblings (see online supplementary table S7), but the mean differences were not significantly different across the average twin mean compared with singleton mean scores except for paranoia (at *P* < .05) although this was not significant if correcting for multiple testing (based on *P* < .01).

#### Outliers.

Based on an outlier definition of scores ±3.5 SD from mean, frequencies of outliers were the following: cognitive disorganization (zero), grandiosity (90), paranoia (76), parent-rated negative symptoms (113), hallucinations (88), and anhedonia (12). In repeated analyses excluding outliers, the data led to the same pattern of results.

### Associations of Psychotic Experiences With Affect and Personality

Table 3 presents correlations with anxiety, depression, and personality. Within self-ratings, cognitive disorganization, paranoia, and hallucinations showed significant positive moderate correlations with both self-rated anxiety sensitivity and self-rated depression (all *r* >.4, *P* < .001). In contrast, anhedonia and grandiosity both showed weak correlations with self-rated anxiety sensitivity and depression (*r* = ±.01–.11), which were all significantly lower than the aforementioned correlations of >.4. Parent-rated negative symptoms showed significant positive moderate correlations with both parent-rated anxiety and depression (both *r* >.4, *P* < .001). Despite overall lower cross-rater correlations between parent ratings and self-ratings, mostly they showed the same pattern in terms of significance and direction of association as the within-rater correlations (see table 3).

**Table 3.** Correlations Between Psychotic Experiences and Anxiety, Depression, and Personality

	Anxiety and Depression				Personality				
	Anxiety Sens	Depression	Anxiety (P)	Depression (P)	Neuroticism	Extraversion	Openness	Agreeableness	Conscientiousness
Paranoia	.40**	.51**	.16**	.20**	.35**	−.12**	.05*	−.15**	−.11**
Hallucinations	.44**	.40**	.14**	.13**	.18**	−.05	.09**	−.09**	−.09**
Cognitive disorganization	.52**	.58**	.25**	.28**	.42**	−.16**	.08**	−.02	−.24**
Grandiosity	.10**	−.00	−.04**	−.03	−.13**	.16**	.12**	−.08**	.11**
Anhedonia	−.11**	.08**	.06**	.07**	.13**	−.27**	−.11**	−.17**	−.12**
Parent-rated negative symptoms	.09**	.19**	.44**	.45**	.15**	−.18**	−.01	−.09**	−.15**

*Note*: *N* = 4701–4741 for correlations with anxiety and depression measures; *N* = 1797–1810 for correlations with personality subscales (a subset of the sample completed the personality subscales). All scales were self-rated except where P (parent-rated) is shown; anxiety sens = anxiety sensitivity.

**P < .001 and *P < .05.

SPEQ and personality scales correlated ±.01–.42. The median correlation was .13, suggesting a minority of variance in psychotic experiences was shared with personality scales. Only 2 correlations were >.3, between neuroticism with paranoia and cognitive disorganization (*r* = .42 and .35, respectively, both *P* < .001).

## Discussion

### Distinct Specific Psychotic Experiences

Our data suggest that specific psychotic experiences appear to be quite distinct: they do not necessarily occur together in a general population sample of 16-year olds. One individual may experience frequent paranoid thoughts without many other experiences, another might experience hallucinations and cognitive disorganization, and another might show only negative symptoms. This implies that if the general criterion for clinical support in adolescents were that of frequent psychotic experiences at a particular time, there would be marked heterogeneity in the type and combination of experiences that these individuals displayed. This study shows that even with truly dimensional quantitative assessments, psychotic experiences appear not to co-occur strongly in adolescence.

It was hypothesized that “positive” SPEQ items would comprise multiple separate components, in line with previous findings,^10–12^ and this was found. The resultant positive SPEQ subscales showed modest to weak correlations with one another. “Negative” SPEQ items fell into the parent-rated negative symptoms and anhedonia components. The SPEQ negative subscales correlated weakly with the positive subscales. The PCA results are comparable to factor analyses of symptoms in samples at high risk for psychosis and with psychotic disorders to the extent that they all present multiple factor solutions^38^ although some differences are apparent. The 2 negative symptom components were somewhat comparable to findings on negative symptoms in schizophrenia, where the separation of diminished expression and anhedonia is the most commonly reported pattern.^20,39^ Unlike previous studies, this study analyzed dimensional assessments of both positive and negative experiences together in adolescence and showed that they separate into distinct components.

### Quantitative Variation in Psychotic Experiences

Categorical assessments of presence/absence of psychotic experiences in adolescence may not reflect the underlying phenotypes, which appear to be continuous in the general population. Specific psychotic experiences in 16-year olds showed considerable quantitative variation, in line with previous reports.^10^ All items were endorsed to some degree by individuals in the sample. Whether psychosis is best considered a quantitative phenotype(s) related to traits or experiences in the general population, or a categorical phenotype, qualitatively distinct to typical development, or something in between—such as discontinuous subpopulations—is much-debated.^4,40^ Empirical data are needed to help ascertain which model is most useful for research and clinical practice. Our data support the proposal that psychotic experiences in the adolescent general population comprise multiple, distinct, quantitative traits. Further longitudinal follow-up of this sample will inform whether these distinct traits become more coherent over time. Compared to previous research on adolescents, the SPEQ measure included a wider range of experiences in its scales. For example, the paranoia subscale asked about both mild suspicions all the way to fears of conspiracies.

### Measurement Properties

The SPEQ is a new questionnaire battery that assesses 6 psychotic experience domains. These domains had good internal consistency and were stable over time. Positive skew, a common feature of measures of psychopathology, was present in 4 of the domains (paranoia, hallucinations, grandiosity, and parent-rated negative symptoms). Validity of the positive SPEQ subscales was supported given their agreement with PLIKS. Females reported more positive psychotic experiences (with exception of grandiosity) and males had higher scores on grandiosity, anhedonia, and parent-rated negative symptoms, which are analogous results to those from CAPE.^10^ Just as there is high comorbidity between adulthood psychosis with affective disorders such as depression and anxiety disorders, there was also a close relationship between SPEQ and affective traits. Variance in paranoia, hallucinations, cognitive disorganization, and parent-rated negative symptoms was shared partly with traits of anxiety, depression, and neuroticism, as reported elsewhere.^7^ SPEQ did not appear to be simply a measure of the main personality dimensions. However, grandiosity and anhedonia shared no or little variance with these traits, suggesting the link with traits of emotional disorders are not present across all specific psychotic experiences (see also Barragan and colleagues).^7^

The anhedonia subscale might have been expected to show a stronger correlation with parent-rated negative symptoms although the use of 2 different raters for the 2 scales will have had the effect of reducing the overall correlation. The correlations between Anhedonia and affective traits were lower than the equivalent correlations for the other SPEQ subscales. It is notable that findings on the CAPE in adolescents reported that of the 3 CAPE negative domains, 1 (affective flattening) was unrelated to depressive symptoms, whereas the other 2 (avolition and social withdrawal) were positively associated.^7^ Together these data suggest that some “negative” psychotic experiences in adolescents may not show as strong a link to affective traits as other forms of psychotic experiences.

Hallucinations and paranoia were assessed in terms of frequency of these experiences, which is standard for these phenomena. Although this study was not designed specifically to assess prevalence of psychotic experiences, some information about frequency is available and can be compared with data from singletons. In our sample, 5.9% reported having auditory vocal hallucinations monthly or more often, which was similar to previous meta- analysis findings on this item.^4,18^ Paranoia was also common although the reported frequencies appeared somewhat lower than in adult samples, where between 3% and 52% of participants reported weekly paranoid thoughts.^21^ The response scales for the remaining 4 subscales were not based on temporal frequency. Nevertheless, the subscale means and frequencies for individual items give an indication of the frequency of these experiences.

### Strengths and Limitations

These data should be considered in light of several limitations. It is important that the findings generalize to singletons. Comparisons of twins and their matched singleton siblings showed, for the most part, no significant differences on the SPEQ. If there are twin-singleton differences, the pattern from our data and other data^41^ suggest twins may show reduced rather than increased levels of psychotic experiences compared with singletons. Past studies also support the assumption that self-report of psychiatric symptoms is equivalent in twins and singletons.^42^ It might also be considered a limitation that the SPEQ did not assess mania. Fewer components for negative symptoms were found compared with the CAPE, which showed separate social withdrawal, affective flattening, and avolition components^7^ although notably the CAPE, unlike SPEQ, relies solely on self-report. Anhedonia did not correlate significantly with cognitive disorganization. Previous work on a sample of help-seeking adults (identified as ultrahigh risk for psychosis several years previously) reported these domains to be on separate factors but in contrast to the present data, reported significant phenotypic correlations between them.^43^ It has been argued previously, given their historical intertwining within psychosis, that these 2 domains are closely linked.^9^ The present data suggest that anhedonia and cognitive disorganization do not show a strong association in adolescent community samples. The majority of SPEQ relies on self-report, which is the modus operandi for assessing psychotic experiences in large samples. Self-report of psychotic experiences has been validated against in-depth clinical interviews^44^ but is known to give higher mean scores than interviews.^4^

The study benefited from having a large, representative sample. This allowed the full range of psychotic experiences to be captured and provided excellent power. A clinical sample was not desired because the purpose was to investigate specific psychotic experiences in the general population of adolescents at an age prior to the typical onset of psychotic disorders.

### Clinical Implications

These results suggest that psychotic experiences in adolescence are highly heterogeneous between individuals even when assessed dimensionally as they were here. Psychotic experiences show high co-occurrence with anxiety and depression, which may act as risk factors for, or exacerbate, psychotic experiences.^45,46^ The high frequency of reported psychotic experiences, in conjunction with the relatively lower frequencies of reported distress levels and relatively lower prevalence of the ultrahigh risk state, suggests a proportion of adolescents have psychotic experiences but remain healthy and do not seek clinical help. Notably, it was cognitive disorganization and paranoia that were associated with the relative greatest levels of distress. Research is needed on psychotic experiences that occur with distress in adolescence, but in addition, research into psychotic experiences that occur without distress may provide clues regarding management and resilience factors in adolescence.^21^

## Supplementary Material

Supplementary material is available at http://schizophre niabulletin.oxfordjournals.org.

## Funding

Medical Research Council (G1100559 to A.R., G0901245, G0500079 to R.P.). D.F. was supported by the Medical Research Council (G0902308); D.S. by the Economic and Social Research Council; C.M.A.H. by the British Academy.

## Supplementary Material

Supplementary Data
