# Supplementary material for: Characterization of Psychotic Experiences in Adolescence Using the Specific Psychotic Experiences Questionnaire: Findings From a Study of 5000 16-Year-Old Twins
Source: Schizophr Bull. 2013 Sep 23;40(4):868–77. doi: 10.1093/schbul/sbt106 (PMC4059437; doi:10.1093/schbul/sbt106)
Supplement: Supplementary Data [file supp_40_4_868__index.html]

Characterization of Psychotic Experiences in Adolescence Using the Specific Psychotic Experiences Questionnaire: Findings From a Study of 5000 16-Year-Old Twins — Characterization of Psychotic Experiences in Adolescence Using the Specific Psychotic Experiences Questionnaire: Findings From a Study of 5000 16-Year-Old Twins — Supplementary Data 

# Characterization of Psychotic Experiences in Adolescence Using the Specific Psychotic Experiences Questionnaire: Findings From a Study of 5000 16-Year-Old Twins

## 

Data files

**Files in this Data Supplement:**

- Supplementary Data - Supplementary Data
