# Supplementary material for: Characterization of Psychotic Experiences in Adolescence Using the Specific Psychotic Experiences Questionnaire: Findings From a Study of 5000 16-Year-Old Twins
Source: Schizophr Bull. 2013 Sep 23;40(4):868–77. doi: 10.1093/schbul/sbt106 (PMC4059437; doi:10.1093/schbul/sbt106)
Supplement: Supplementary Data [file supp_sbt106_SupplementaryMat_R2.doc]

Supplementary Material

Appendix

*Specific Psychotic Experiences Questionnaire (SPEQ)*

The SPEQ was developed principally by selecting and combining items from existing scales for adults, adapting wording and items when necessary. Age appropriateness of items was ensured via piloting on 17-year-old singletons (see description of the pilot sample in the *Methods*) and via expert clinical opinion. Six types of psychotic experiences were measured. Paranoia, Hallucinations, Cognitive Disorganization, Grandiosity, Anhedonia were assessed via self report while further Negative Symptoms were assessed via parent report.

*Paranoia*

*Paranoia* was assessed with 15 items from the Paranoia Checklist. Participants were asked to rate how often they had each thought or feeling on a 6-point scale: “*Not at all”* (0)*,“Rarely”* (1)*, “Once a month”* (2)*,“Once a week”* (3)*, “Several times a week”* (4)*, “Daily”* (5)*.* From the original 19-item Paranoid Checklist (Freeman et al, 2005), seven items were reworded for clarity for the age group (detailed below), in one instance, two similar items were combined into one item (“Someone I know has bad intentions towards me" and "Someone I don’t know has bad intentions towards me" combined into one item "Someone has bad intentions towards me"), and three items were omitted (“People communicate about me in subtle ways”, “People would harm me if given an opportunity”, “My actions and thoughts might be controlled by others”). Wording changes were introduced to seven items from Paranoid Checklist (“There might be negative comments being circulated about me" changed to "There might be negative comments being spread about me"; “People deliberately try to irritate me” changed to “People are deliberately trying to irritate me”; “People are trying to make me upset" changed to "People are trying to upset me"; "Strangers and friends look at me critically" changed to "People are looking at me in an unfriendly way”; "People might be hostile towards me" changed to "People are being hostile towards me"; "I have a suspicion that someone has it in for me" changed to "Someone has it in for me"; "There is a possibility of a conspiracy against me" changed to "People might be conspiring against me").

*Hallucinations*

*Hallucinations* were assessed with nine items selected from the Cardiff Anomalous Perceptions Scale (CAPS; Bell et al 2005). Participants were asked how frequently they had each of the experiences using the rating scale: “*Not at all”* (0)*, “Rarely”* (1)*, “Once a Month”* (2)*, “Once a Week”* (3), *“Several Times a Week”* (4)*, “Daily”* (5) as per the published instrument. A representative selection of items were chosen from the CAPS measure to capture the main different types of sensory hallucinations: sight (2 items), touch (2 items), sound (2 items), sound-vocal (1 item) and smell (2 items).

*Cognitive Disorganization*

*Cognitive Disorganization* was assessed with 11 items from the Cognitive Disorganization subscale of the short Oxford-Liverpool Inventory of Feelings and Experiences [^26^](#_ENREF_26). Participants were asked to base their responses on their feelings over the last month. Items had “*yes*”(1) “*no*”(0) responses as per the published instrument. No changes were made to the original item wording. Items tapped aspects of poor concentration and attention, difficulty with decision-making and social anxiety. These facets are conceptually linked to formal thought disorder and other disorganised aspects of psychosis.

*Grandiosity*

*Grandiosity* was assessed with eight items: three from the “Myself” sub-scale of Cognition Checklist for Mania-Revised (CCL-M-R; Beck, 2006), two items from the Peters et al. Delusions Inventory (PDI; [Peters et al](#_ENREF_28)), and three items were developed from clinical case studies. Participants were asked how much they agreed with each statement, based on their thoughts and feelings over the last month. Responses had a 4-point scale: “*Not at all*” (0), “*Somewhat*” (1), “*A great deal*” (2), “*Completely*” (3) as per the CCL-M-R (Beck et al., 2006).

In developing the scale, the seven-item “Myself” subscale of CCL-M-R was piloted on 17-year-olds (see *Methods* section for pilot sample details). Feedback from the pilot and analysis of the frequency of endorsement suggested that four items from this scale were not suitable for a general population non-clinical sample of adolescents because they could be interpreted in multiple ways (e.g. “I am strong”, “I am the best”) or caused confusion in a non-clinical setting (“People must hear my message”, “Good things are in store for me”). Therefore, following the pilot, three items from the “Myself” subscale measure were retained (“I have a special mission”; “I have many great ideas”;” Everything I do is great”) and four items were dropped. Two items were added from the PDI (Peters et al, 2004) that capture believing self to be someone special (“Do you ever feel as if you are, or destined to be someone very important?” changed to “I am, or am destined to be someone very important”, and “Do you ever feel that you are a very special or unusual person?” changed to “I am a very special or unusual person”). Finally, three items were added that had been developed by DF based on clinical case notes of patients with grandiose delusions (“I have special abilities that others do not”; “I am much more unique than everyone else”; “Everyone is going to know about me because of my greatness”).

*Anhedonia*

*Anhedonia* was assessed with ten items from the anticipatory pleasure subscale of the Temporal Experience of Pleasure Scale (Gard et al., 2006). Participants were asked to base their ratings on their thoughts and feelings over the last month. Items were rated on a 6-point scale: “V*ery false for me”* (0)*, “Moderately false for me”* (1)*, “Slightly false for me”* (2)*, “Slightly true for me”* (3)*, “Moderately true for me”* (4)*, “Very true for me*” (5)*,* as per the original instrument. No changes were made to the original item wording. The scale was reversed so that higher scores indicated more Anhedonia.

*Parent-rated Negative Symptoms*

*Parent-rated Negative Symptoms* were assessed with ten items devised based on the content of the Scale for the Assessment of Negative Symptoms (SANS; Andreasen, 1984). Items were rated on a 4-point scale: ‘*Not at all true”* (0), *“Somewhat true”* (1), *“Mainly true”* (2), *“Definitely true*” (3). Parents were asked to rate how strongly they agreed with each statement. Items tapped into five SANS areas: affective flattening or blunting, alogia, avolition-apathy, anhedonia-asociality, and attention.

*Distress*

Finally, *distress* was assessed with an item at the end of each of the Paranoia, Cognitive Disorganization, Grandiosity and Hallucinations subscales: “*Overall, how distressed are you by these experiences?”* with the scale “*Not distressed”, “A bit distressed”, “Quite distressed”, “Very distressed*”*.*

Table S1. Demographic information and behavior problem scores for participating and non-participating families in the LEAP study.

|  | Participating in LEAP | | Non-participating in LEAP | |  |
| --- | --- | --- | --- | --- | --- |
| Male | 45% |  | 53% |  |  |
| Monozygotic | 35% |  | 32% |  |  |
| White | 94% |  | 91% |  |  |
| Mothers had one or more A-levels (UK advanced educational qualification) as highest qualification | 16% |  | 12% |  |  |
|  | M | SD | M | SD | *p* |
| SDQ Total scale, age 4 years | 8.54 | 4.44 | 9.41 | 4.70 | <.001 |
| SDQ Emotional problems subscale, age 4 years | 1.33 | 1.41 | 1.46 | 1.50 | <.001 |
| SDQ Total scale, age 12 years | 6.80 | 5.03 | 7.91 | 5.44 | <.001 |
| SDQ Emotional problems subscale, age 12 years | 1.81 | 1.91 | 1.93 | 2.03 | <.05 |

Note. SDQ, Strengths and Difficulties Questionnaire.

Table S2. Principal component analysis loadings for the six extracted components using Varimax rotation

|  | Component 1: Paranoia | Component 2: Hallucinations | Component 3: Parent-rated Negative Symptoms | Component 4: Grandiosity | Component 5: Cognitive Disorganization | Component 6: Anhedonia^a^ |
| --- | --- | --- | --- | --- | --- | --- |
| Variance explained/Items | 12.47% | 7.29% | 7.02% | 6.53% | 5.71% | 5.61% |
| Someone has bad intentions towards me | .80 |  |  |  |  |  |
| Bad things are being said about me behind my back | .79 |  |  |  |  |  |
| People are being hostile towards me | .78 |  |  |  |  |  |
| People are trying to upset me | .77 |  |  |  |  |  |
| Someone has it in for me | .75 |  |  |  |  |  |
| People are looking at me in an unfriendly way | .74 |  |  |  |  |  |
| There might be negative comments being spread about me | .73 |  |  |  |  |  |
| People might be conspiring against me | .71 |  |  |  |  |  |
| I am under threat from others | .70 |  |  |  |  |  |
| People are laughing at me | .70 |  |  |  |  |  |
| People would harm me if given an opportunity | .69 |  |  |  |  |  |
| People are deliberately trying to irritate me | .63 |  |  |  |  |  |
| I need to be on my guard against others | .61 |  |  |  |  |  |
| I might be being observed or followed | .52 |  |  |  |  |  |
| I can detect coded messages about me in the press/TV/internet | .38 |  |  |  |  |  |
| Hear sounds or music that people near you don’t hear? |  | .72 |  |  |  |  |
| See things that other people cannot? |  | .72 |  |  |  |  |
| Feel that someone is touching you, but when you look nobody is there? |  | .70 |  |  |  |  |
| Hear noises or sounds when there is nothing about to explain them? |  | .68 |  |  |  |  |
| Detect smells which don’t seem to come from your surroundings? |  | .68 |  |  |  |  |
| See shapes, lights, or colors even though there is nothing really there? |  | .67 |  |  |  |  |
| Notice smells or odors that people next to you seem unaware of? |  | .65 |  |  |  |  |
| Experience unusual burning sensations or other strange feelings in or on your body that can’t be explained? |  | .59 |  |  |  |  |
| Hear voices commenting on what you’re thinking or doing? |  | .58 |  |  |  |  |
| Has a lack of energy and motivation (p) |  |  | .74 |  |  |  |
| Often does not have much to say for himself/herself (p) |  |  | .70 |  |  |  |
| Usually gives brief, one word replies to questions, even if encouraged to say more (p) |  |  | .69 |  |  |  |
| Is often inattentive and appears distracted (p) |  |  | .68 |  |  |  |
| Seems emotionally “flat”, for example, rarely changes the emotions he/she shows (p) |  |  | .67 |  |  |  |
| Often sits around for a long time doing nothing (p) |  |  | .66 |  |  |  |
| Has very few interests or hobbies (p) |  |  | .66 |  |  |  |
| Often does not pay attention when being spoken to (p) |  |  | .65 |  |  |  |
| Often fails to smile or laugh at things others would find funny (p) |  |  | .55 |  |  |  |
| Has few or no friends (p) |  |  | .50 |  |  |  |
| I am, or am destined to be, someone very important |  |  |  | .78 |  |  |
| Everyone is going to know about me because of my greatness |  |  |  | .75 |  |  |
| I am a very special or unusual person |  |  |  | .73 |  |  |
| I am much more unique than anyone else |  |  |  | .71 |  |  |
| I have special abilities that others do not |  |  |  | .71 |  |  |
| Everything I do is great |  |  |  | .70 |  |  |
| I have many great ideas |  |  |  | .66 |  |  |
| I have a special mission |  |  |  | .53 |  |  |
| Are you easily confused if too much happens at the same time? |  |  |  |  | .61 |  |
| Are you easily distracted when you read or talk to someone? |  |  |  |  | .61 |  |
| Do you frequently have difficulty in starting to do things? |  |  |  |  | .58 |  |
| Do you find it difficult to keep interested in the same thing for a long time? |  |  |  |  | .54 |  |
| Are you easily distracted from work by daydreams? |  |  |  |  | .53 |  |
| When in a crowded room, do you often have difficulty in following a conversation? |  |  |  |  | .52 |  |
| Is it hard for you to make decisions? |  |  |  |  | .51 |  |
| Do you often have difficulties in controlling your thoughts? |  |  |  |  | .49 |  |
| Do you ever feel that your speech is difficult to understand because the words are all mixed up and don’t make sense? |  |  |  |  | .45 |  |
| Are you a person whose mood goes up and down easily? |  |  |  |  | .43 |  |
| Do you dread going into a room by yourself where other people have already gathered and are talking? |  |  |  |  | .41 |  |
| When ordering something off a menu, I imagine how good it will taste |  |  |  |  |  | .67 |
| Looking forward to a pleasurable experience is in itself pleasurable |  |  |  |  |  | .66 |
| When I think about eating my favorite food, I can almost taste how good it is |  |  |  |  |  | .63 |
| I get so excited the night before a major holiday I can hardly sleep |  |  |  |  |  | .63 |
| When something exciting is coming up in my life, I really look forward to it |  |  |  |  |  | .61 |
| I look forward to a lot of things in my life |  |  |  |  |  | .60 |
| When I hear about a new movie starring my favorite actor, I can’t wait to see it |  |  |  |  |  | .58 |
| When I think of something tasty, like a chocolate biscuit, I have to have one |  |  |  |  |  | .54 |
| When I’m on my way to an amusement park, I can hardly wait to ride the roller coaster  I don’t look forward to things like eating out at restaurants (R) |  |  |  |  |  | .46  .37 |

Note. All items were self-report except where indicated as parent-report (p). Bartlett’s test of sphericity indicated that correlations between items were sufficiently large for PCA (X2 (1953) = 99951.32, p<0.001). ^a^Original (unreversed) items from the Anhedonia scale were used in the PCA.

Kaiser-Meyer-Olkin (KMO) value of .93 revealed that the sample size was highly desirable for these analyses. This was further supported by anti-image values all > .8. Average communality in variables was .45.

Table S3. Frequencies for distress items.

|  | *Not distressed* | *A bit distressed* | *Quite distressed* | *Very distressed* | No response |
| --- | --- | --- | --- | --- | --- |
| Paranoia | 73.2% | 20.2% | 3.6% | 0.6% | 2.4% |
| Hallucinations | 79.9% | 9.4% | 1.3% | 0.4% | 9.1% |
| Cognitive Disorganization | 74.4% | 19.2% | 4.8% | 0.9% | 0.8% |
| Grandiosity | 87.5% | 9.2% | 1.3% | 0.4% | 1.7% |

Note. *N* = 3344-3650. The reduced sample size compared to the other data is due to the distress items being included for only a subsample of the total LEAP sample.

Table S4. Mean sex differences.

|  | Male |  | Female |  |  |  |  |  |
| --- | --- | --- | --- | --- | --- | --- | --- | --- |
|  | M | SD | M | SD | *t* | *df* | *p* | *d* |
| Paranoia | 11.76 | 10.42 | 12.45 | 10.70 | 2.86 | 1, 4444.03 | .004 | .08 |
| Hallucinations | 4.29 | 5.77 | 4.89 | 6.06 | 4.28 | 1, 4737 | < .001 | .12 |
| Cognitive Disorganization | 3.40 | 2.73 | 4.39 | 2.87 | 12.13 | 1,4609.60 | <.001 | .35 |
| Grandiosity | 5.82 | 4.56 | 4.89 | 4.25 | 7.77 | 1, 4600.12 | <.001 | .23 |
| Anhedonia | 18.53 | 7.99 | 14.60 | 7.43 | 17.34 | 1, 4377.94 | <.001 | .51 |
| Parent-rated Negative Symptoms | 3.18 | 4.11 | 2.53 | 3.69 | 7.41 | 1, 4744 | <.001 | .22 |

*Note*. SD = standard deviations; *df* = degrees of freedom. Raw scores provided. Paranoia, Hallucinations, Grandiosity and Parent-rated Negative Symptoms were transformed prior to statistical testing.

Table S5. Correlations between psychotic experiences and with anxiety, depression and personality, split by sex.

|  | Pr | Ha | Cg | Gr | An | Anx (S) | Anx (P) | Dep (S) | Dep (P) | Neu | Ext | Op | Ag | Con |
| --- | --- | --- | --- | --- | --- | --- | --- | --- | --- | --- | --- | --- | --- | --- |
| Pr |  |  |  |  |  | .43**/ | .19**/ | .54**/ | .24**/ | .38**/ | -.16**/ | .05/ | -.16**/ | -.13**/ |
|  |  |  |  |  |  | .39** | .12** | .47** | .15** | .31** | -.06 | .04 | -.15** | -.07 |
| Ha | .46**/ |  |  |  |  | .45**/ | .16**/ | .42**/ | .15**/ | .23**/ | -.09**/ | .06/ | -.12**/ | -.16**/ |
|  | .44** |  |  |  |  | .42** | .10** | .37** | .10** | .12** | .01 | .12** | -.06 | -.01 |
| Cg | .43**/ | .45**/ |  |  |  | .50** / | .24**/ | .56**/ | .28**/ | .44**/ | -.19**/ | .07*/ | -.11**/ | -.29**/ |
|  | .38** | .41** |  |  |  | .49** | .19** | .56** | .25** | .34** | -.14** | .08* | .03 | -.21** |
| Gr | .07**/ | .16**/ | -.01/ |  |  | .10**/ | -.03 | -.03/ | -.04*/ | -.18**/ | .20**/ | .11**/ | -.07*/ | .11**/ |
|  | .16** | .23** | .09** |  |  | .17** | .00 | .08** | .01 | -.03 | .13** | .15** | -.02 | .15** |
| An | .09**/ | .03/ | .07*/ | -.21**/ |  | -.02/ | .12**/ | .16**/ | .10**/ | .18**/ | -.25**/ | -.12** | -.13**/ | -.08**/ |
|  | .06* | -.01 | .05* | -.20** |  | -.06** | .11** | .12** | .10** | .14** | -.27** | -.10** | -.10** | -.11** |
| NS | .16**/ | .18**/ | .27**/ | -.03/ | .11**/ | .15**/ | .49**/ | .24**/ | .46**/ | .19**/ | -.18**/ | -.03/ | -.05/ | -.16**/ |
|  | .12** | .10** | .24** | -.02 | .11** | .11** | .46** | .18** | .45** | .13** | -.16** | .03 | -.08* | -.12** |

Note. Male/ female correlations presented. Pr=Paranoia; Ha=Hallucinations; Cg=Cognitive Disorganization; An=Anhedonia; Gr=Grandiosity; NS=Parent-rated Negative Symptoms; Anx (S)=Anxiety sensitivity self-report; Anx (P)=Anxiety parent-report; Dep (S)=Depression self-report; Dep (P)=Depression parent-report; Neu=Neuroticism; Ext=Extraversion; Op=Openness; Ag=Agreeableness; Con=Conscientiousness. Approximate *N* (males/females) = 2124*/*2619, except for correlations with personality where approximate *N* (males/females) = 720/1069. ** *p* < .001; **p* < .05.

Table S6. Mean differences on specific psychotic experiences between individuals with and without at least one self-endorsed psychotic symptom.

|  | PLIKS=0  (N = 1118-1129) | | PLIKS=1  (N = 186-188) | |  |  |  |  |
| --- | --- | --- | --- | --- | --- | --- | --- | --- |
|  | M | SD | M | SD | *t* | *df* | *p* |  |
| Paranoia | 12.65 | 12.05 | 28.17 | 17.37 | 14.27 | 294.14 | <.001 |  |
| Hallucinations | 5.38 | 6.13 | 14.36 | 9.90 | 15.41 | 279.34 | <.001 |  |
| Cognitive Disorganization | 3.43 | 4.44 | 5.01 | 5.69 | 10.04 | 1314 | <.001 |  |
| Grandiosity | 5.38 | 6.13 | 14.36 | 9.90 | 6.69 | 11314 | <.001 |  |
| Anhedonia | 17.82 | 7.98 | 18.26 | 8.60 | 0.70 | 1315 | .48 |  |
| Parent-rated Negative Symptoms | 4.29 | 4.43 | 7.22 | 6.04 | 4.18 | 1302 | <.001 |  |

*Note*. SD, standard deviations; *df*, degrees of freedom. PLIKS, Psychosis-Like Symptoms self-report questionnaire. SPEQ Paranoia, Hallucinations, Grandiosity and Parent-rated Negative Symptoms subscales were transformed prior to statistical testing. PLIKS data were collected in LEAP Phase 2 sample. SPEQ raw scores are provided.

Table S7. Mean differences between twins and singleton siblings on psychotic experiences.

|  | Mean | SD | N | *t* | *df* | *p* |  |
| --- | --- | --- | --- | --- | --- | --- | --- |
| Paranoia Twin 1 | 11.02 | 9.90 | 37 | 2.82 | 36 | .008 |  |
| Paranoia Twin 2 | 11.21 | 7.52 | 37 | 2.20 | 36 | .035 |  |
| Paranoia Twins’ mean | 11.12 | 8.71 |  |  |  |  |  |
| Paranoia Sibling | 17.09 | 11.93 | 44 |  |  |  |  |
| Hallucinations Twin 1 | 3.38 | 5.51 | 37 | 4.33 | 36 | .000 |  |
| Hallucinations Twin 2 | 5.46 | 5.85 | 37 | .905 | 36 | .371 |  |
| Hallucinations Sibling | 6.09 | 5.86 | 45 |  |  |  |  |
| Cognitive Disorganization Twin 1 | 3.23 | 3.09 | 37 | 1.65 | 36 | .109 |  |
| Cognitive Disorganization Twin 2 | 4.00 | 3.10 | 37 | 0.01 | 36 | .991 |  |
| Cognitive Disorganization Sibling | 4.22 | 2.71 | 45 |  |  |  |  |
| Anhedonia Twin 1 | 18.19 | 8.04 | 37 | 1.30 | 36 | .202 |  |
| Anhedonia Twin 2 | 19.64 | 8.15 | 37 | 2.70 | 36 | .010 |  |
| Anhedonia Sibling | 16.18 | 9.92 | 45 |  |  |  |  |
| Parent-rated Negative Symptoms Twin 1 | 2.40 | 3.36 | 37 | 0.68 | 36 | .502 |  |
| Parent-rated Negative Symptoms Twin 2 | 2.08 | 3.12 | 36 | 1.17 | 35 | .249 |  |
| Parent-rated Negative Symptoms Sibling | 2.82 | 3.68 | 45 |  |  |  |  |

*Note*. SD, standard deviations; *df*, degrees of freedom. Grandiosity not included due to lack of equivalent sibling data. Paranoia, Hallucinations and Parent-rated Negative Symptoms were transformed prior to statistical testing. Raw scores provided. Data on singletons came from pilot study (see *Methods*).
